# Reproducibility of a 3-dimensional gyroscope in measuring shoulder anteflexion and abduction

**DOI:** 10.1186/1471-2474-13-135

**Published:** 2012-07-30

**Authors:** Ludo I F Penning, Nick A Guldemond, Rob A de Bie, GeertHIM Walenkamp

**Affiliations:** 1 Department of Orthopaedic Surgery, Maastricht University Medical Centre, Research, P. Debeyeplein 25, PO Box 5800, 6202 AZ, Maastricht, The Netherlands; 2 Research Institute Caphri, Maastricht University, PO Box 616, 6200 MD, Maastricht, The Netherlands; 3 Delft University of technology, PO Box 5046, 2600 GA, Delft, The Netherlands; 4 Department of Epidemiology Maastricht University, Research institute Caphri, P.Debeyeplein 1, PO Box 616, 6200 MD, Maastricht, The Netherlands

**Keywords:** Reproducibility, Shoulder, Tri axial gyroscope, Range of motion, Shoulder function, Generalizability theory

## Abstract

**Background:**

Few studies have investigated the use of a 3-dimensional gyroscope for measuring the range of motion (ROM) in the impaired shoulder. Reproducibility of digital inclinometer and visual estimation is poor. This study aims to investigate the reproducibility of a tri axial gyroscope in measurement of anteflexion, abduction and related rotations in the impaired shoulder.

**Methods:**

Fifty-eight patients with either subacromial impingement (27) or osteoarthritis of the shoulder (31) participated. Active anteflexion, abduction and related rotations were measured with a tri axial gyroscope according to a test retest protocol. Severity of shoulder impairment and patient perceived pain were assessed by the Disability of Arm Shoulder and Hand score (DASH) and the Visual Analogue Scale (VAS). VAS scores were recorded before and after testing**.**

**Results:**

In two out of three hospitals patients with osteoarthritis (n = 31) were measured, in the third hospital patients with subacromial impingement (n = 27).

There were significant differences among hospitals for the VAS and DASH scores measured before and after testing. The mean differences between the test and retest means for anteflexion were −6 degrees (affected side), 9 (contralateral side) and for abduction 15 degrees (affected side) and 10 degrees (contralateral side).

Bland & Altman plots showed that the confidence intervals for the mean differences fall within −6 up to 15 degrees, individual test - retest differences could exceed these limits.

A simulation according to ‘Generalizability Theory’ produces very good coefficients for anteflexion and related rotation as a comprehensive measure of reproducibility. Optimal reproducibility is achieved with 2 repetitions for anteflexion.

**Conclusions:**

Measurements were influenced by patient perceived pain. Differences in VAS and DASH might be explained by different underlying pathology. These differences in shoulder pathology however did not alter the reproducibility of testing. The use of a tri axial gyroscope is a simple non invasive and reproducible method for the recording of shoulder anteflexion and abduction. Movements have to be repeated twice for reproducible results.

## Background

Range of motion (ROM) active and passive is an essential measure in the diagnosis and evaluation of shoulder impairments [1]. The reproducibility of estimated ROM however, is under discussion and depends on the method used for measurement [2,3]. Several methods have been developed for the measurement of ROM. The techniques range from visual estimation and goniometric measurements to electromagnetic tracking systems, accelerometers and invasive techniques, with sensors mounted on the scapula [4-10]. Most of these measurement techniques have to be performed in a laboratory setting and consist of the placement of multiple sensors on bony landmarks. Placement of sensors on bony landmarks lack a direct bony contact because of overlying tissue and thus could affect the reproducibility of results [11]. The accurate evaluation of ROM of the shoulder is important for clinical decision making and thus should be reproducible. Most exact measurement techniques are time consuming and therefore cannot be performed in the outpatient clinic. Reproducibility of digital inclinometer and visual estimation of range of motion is poor [12,13]. The technique of a tri axial gyroscope could be a quick and simple method for the recording of three-dimensional shoulder movements. In this study we investigated the reproducibility of a tri axial gyroscope to assess active anteflexion and abduction movements in patients with shoulder complaints. Anteflexion and abduction consist of a combined upward movement and related rotation in either frontal or sagital plane. Using the tri axial gyroscope we were able to measure both flexion or abduction and related rotations during movement. *The focus of the study was directed on the assessment of the clinical reproducibility in impaired shoulders, caused by different underlying pathology. The main research questions consisted of the reproducibility of measurement through use of an over-the-counter and the number of times measurements have to be repeated.*

## Methods

### Setting and participants

**Figure 1:**
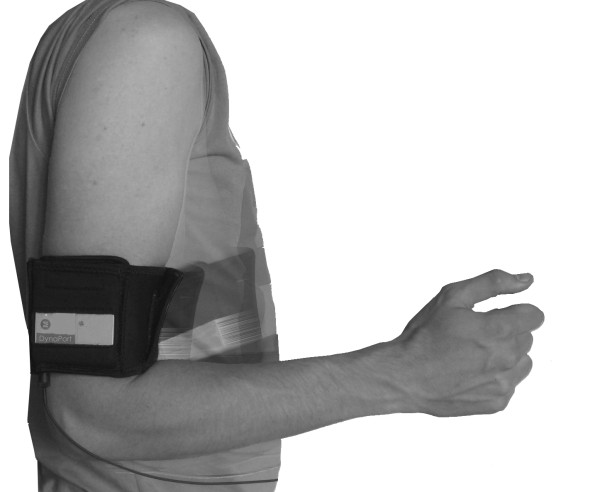
Fixation of Minimod device on upper arm with neoprene strap.

From May 2007 until October 2007 a comprehensive reproducibility study was performed among fifty eight patients suffering from shoulder complaints. Patients were recruited in three hospitals. The underlying shoulder pathology consisted either of subacromial impingement syndrome (n = 27) or osteoarthritis (n = 31). In one hospital patients with subacromial impingement were measured in the other two hospitals patients with osteoarthritis. All patients were awaiting surgery for their complaints. Nine patients suffered from bilateral complaints. The dominant side of these patients was presumed to be the affected side. Patients underwent examination with a Minimod® device (Mc Roberts, The Hague, Netherlands) (Figure 1) for both active anteflexion and abduction and related rotations of their shoulder. The reproducibility of the test was examined according to a test - retest protocol. The local Ethics Committee approved the study protocol and the study was carried out according to the Declaration of Helsinki. *Informed consent was obtained of patients preceding the study.*

### Measurement techniques

*The Minimod 3-dimensional gyroscope is a small box (62 × 41 × 18 mm, 53 grams) containing three gyroscopic sensors. A gyroscope is a device for measuring or maintaining orientation, based on the principles of conservation of an angular momentum*[14]. Data are collected and stored on a Secure Digital (SD) card. In this study both maximum active anteflexion and abduction and related rotations of the shoulder were measured according to a pre defined test protocol.

In order to prevent influence of thoracic spine movement during measurement, the patient was placed with the opposite shoulder against a wall. The Minimod device was fixed with a neoprene strap to the lateral middle part of the upper arm of the patient. (Figure 1) Preceding the actual measurement the Minimod was calibrated for abduction and anteflexion movement. *For the calibration the device was fixed with the neoprene strap. The arm of the patient was first slowly moved in an anteflexion direction by the researcher up to 45 degrees, this movement was recorded with the minimod device. After the anteflexion movement an abduction movement was performed in the same way.* The calibration was used for setting of the x y and z axis needed for further calculation. Each test consisted of five consecutive movements. The start and end of each test was marked by pressing a switch connected to the device. Calibration was performed before each test. After this calibration patients were asked to perform a maximal possible active anteflexion movement for five times. A new calibration was performed before measurement of the abduction movement. Patients were now asked to perform a maximal active abduction for five times, than the contralateral side was measured in the same way for both anteflexion and abduction. During a break of approximately 30 minutes patients completed the DASH score, than a retest was performed for anteflexion and abduction in the affected and contralateral side. A total of 40 (20 per shoulder) movements were recorded for each patient. In order to assess the effect of patient perceived pain on movements, VAS scores were recorded immediately before and after measurement of the test and the retest. Measurements were performed in 3 hospitals by one local (trained) outcome assessor.

### Analysis

Demographic data were collected on age, sex, dominance, length and weight. (Table 1)

**Table 1:** Patient characteristics

	**Hospital 1 (n = 9)**	**SD**	**Hospital 2 (n = 22)**	**SD**	**Hospital 3 (n = 27)**	**SD**	**p-value**
Age	55,9	15,8	55,5	4,1	52,4	7,9	0,70
Lenght	1,7	0,1	1,7	0	1,7	0,1	0,24
Weight	80,1	10,6	74,8	3,5	75,6	14,6	0,66
BMI	26,4	3,3	24,7	0,8	26,5	4,2	0,27
DASH	36,3	23,5	27,1	5,2	48,4	20,6	0,01
Male	4		13		10		0,40
Female	5		9		17		0,40
Affected side left	3		7		8		0,82
Affected side right	6		15		19		0,40
Affected side both	1		6		2		0,38
Dominant side left	1		0		1		0,55
Dominant side right	8		22		26		0,55
Affected side							
Mean VAS score before test	2,74	2,35	3,20	2,98	5,16	3,19	0,03
Mean VAS score after test	2,97	2,39	3,57	3,22	6,07	3,36	0,01
Mean VAS score before retest	3,47	2,89	3,18	3,00	5,33	3,37	0,05
Mean VAS score after retest	3,41	2,49	3,53	3,19	6,61	3,04	0,00
Mean difference VAS score test	0,22	0,51	0,37	0,62	0,91	1,44	0,13
Mean difference VAS score retest	−0,06	1,45	0,35	0,78	1,28	1,57	0,01
Contralateral side							
Mean VAS score before test	0,54	0,56	0,56	1,03	1,69	2,2	0,04
Mean VAS score after test	0,63	0,55	0,58	1,06	2,01	2,3	0,01
Mean VAS score before retest	0,60	0,57	0,66	1,32	1,71	2,1	0,06
Mean VAS score after retest	0,56	0,55	0,74	1,37	2,35	2,4	0,01
mean difference VAS score test	0,03	0,13	0,01	0,12	0,32	0,89	0,20
mean difference VAS score retest	−0,04	0,05	0,08	0,33	0,64	1,41	0,08

*Primary outcome measurements* in this study are the data collected with the Minimod. The data collected with the Minimod consist of the accelerations in (meter/second squared) measured by seismic acceleration sensors in three axis. The raw signals stored on the SD card were exported to ASCI files through use of the MiRA (MiniMod Read and Acquire) software. ASCI files were send to McRoberts, among with a list of the order in which abduction and anteflexion were performed, for further analysis using Matlab® and calculating range of motion in degrees of anteflexion, abduction and related rotations using matrix algebra and goniometric operations*. For the sake of company confidentiality we cannot further explain the method of analysis in Matlab.*

*Secondary outcome measurements* are the VAS scores (Visual Analogue Scale) and the DASH. (Disability of Arm Shoulder and Hand) [15]. The VAS scores were recorded preceding and immediate following each time the Test or Retest was performed. VAS scores were used to examine possible bias in test results caused by patient perceived pain performing the test. Following the first assessment the DASH questionnaire was filled out.

The *Visual Analogue Scale* is a line of 10 cm in length, which is taken to represent the continuum of experienced pain. It has been proved to be a simple, sensitive and reproducible instrument that enables the patients to express their pain in such a way that it can be given a numerical value [16].

### Statistical analysis

The results of individual movements were expressed in degrees of maximum achieved anteflexion or abduction and related rotations. For the analysis of primary outcome results of Test and Retest, the mean of five consecutive movements was calculated, together with the Standard Deviation (SD) and 95% confidence intervals.

Mean differences in degrees between mean results of Test and Retest were calculated.

Bland Altman plots were used to show the level of agreement for Testing and Retesting for the different hospitals. (MedCalc® version 11.3.1.0)

A simulation according to the ‘Generalizability Theory’ was performed for the Test and Retest by combining either anteflexion or abduction and related rotation in a 1-faceted crossed design (P*F1). P represents the total number of persons and F1 represents the 10 level 1-faceted design. The results of this simulation are expressed as generalizability-coefficient (g-coefficient): range 0–1. A g-coefficient of 0,8 is generally accepted as a good reliability ref Shavelson R., Webb N.M. Generalizability Theory: a primer. Measurement methods for the social sciences series 1. Vol. XIII, 1991.

After performing a G-study (generalizability study) also a D-study (decision-study) was performed. By performing a D-study the number of repetitions of measurement for a reliable result can be calculated [17,18].

For the secondary outcome results, DASH and recorded VAS scores before and after testing of the affected side were analyzed using an ANOVA (SPSS 17.0).

## Results

The recruited 58 patients performed a total of 10 movements for each Test. Measuring both affected and contralateral side in a Test and Retest, this resulted in a total of 1160 recordings of anteflexion and 1160 recordings of abduction. The matrix algebra and goniometric operations performed by Mc Roberts resulted in 4640 calculated measures of range of motion, depicted in degrees of anteflexion, abduction and related rotations.

In hospital 1 and 2 patients with osteoarthritis were measured, in hospital 3 patients with subacromial impingement. The recorded patient characteristics were significantly different concerning the DASH score. The score of hospital 3 was significantly higher than the score of hospital 2 these differences can be explained because of different shoulder pathology in the groups. The VAS scores measured preceding and following each test showed significant differences between the participating hospitals for the VAS scores measured before and after the test and after the retest of both affected and contra lateral sides. The mean difference between pre and post test VAS score was significantly different for the retest, VAS scores measured for the contra lateral side did not show significant differences between mean differences of pre and post test VAS scores (Table 1).

The mean differences between the test and retest means for anteflexion were −6 degrees (affected side), 9 (contralateral side) and for abduction 15 degrees (affected side) and 10 degrees (contralateral side) (Table 2).

**Table 2:** Mean and mean differences between test and retest means

	**Anteflexion**		**Ante flexion rotation**		**Abduction**		**Abduction rotation**	
***affected***	**test**	**retest**	**test**	**retest**	**test**	**retest**	**test**	**retest**
mean	149,35	140,37	57,61	51,19	128,75	118,05	−46,43	−46,42
SD	25,61	27,41	37,52	33,33	31,03	35,23	55,10	53,43
mean difference between test and retest (SD)	−6,21	(45,16)	−4,89	(41,39)	15,10	(33,60)	−1,38	(39,82)
*contralatera*								
mean	135,94	128,73	48,65	51,96	118,13	103,45	−34,82	−34,47
SD	40,68	32,10	42,22	25,80	37,43	40,83	52,64	51,49
mean difference between test and retest (SD)	9,41	(29,55)	8,01	(35,66)	10,27	(36,41)	1,02	(35,37)

**Figure 2:**
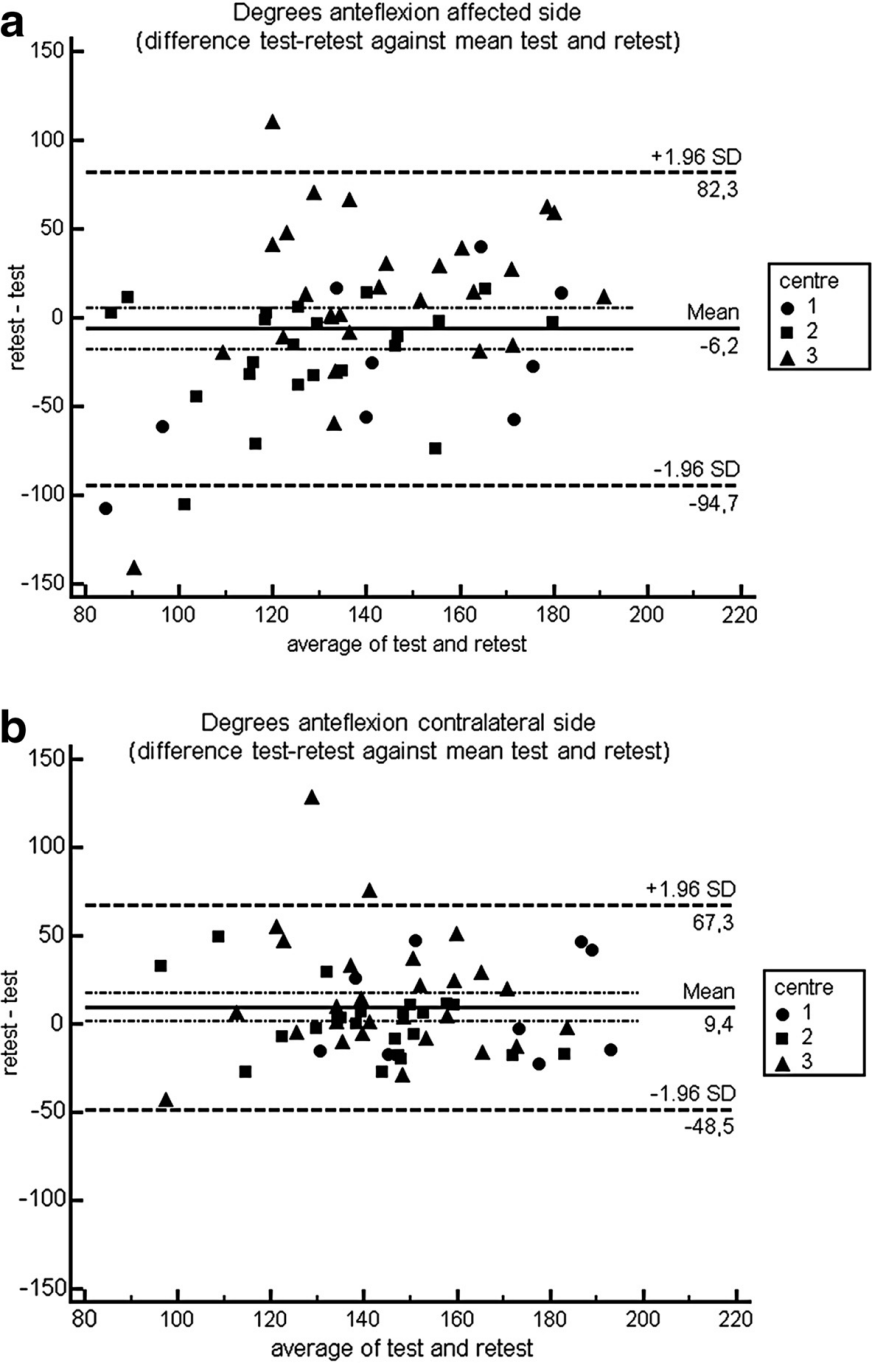
Bland Altman plot anteflexion.

**Figure 3:**
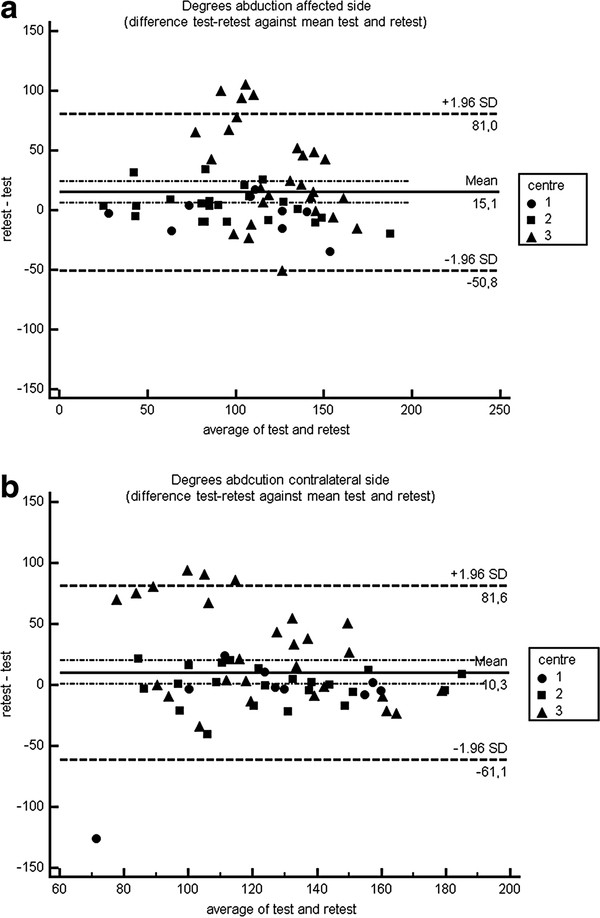
Bland Altman plot abduction.

**Figure 4:**
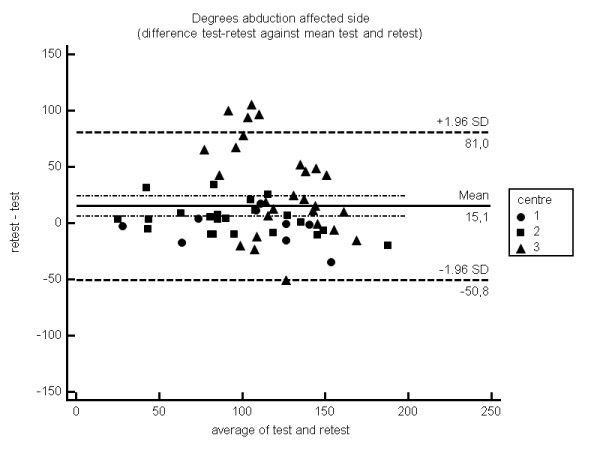
D-study results.

The Bland – Altman plots show individual differences between test and retest measurement regarding anteflexion and abduction for each centre. The confidence intervals for the overall mean differences fall within −6 up to 15 degrees, but individual test-retest differences (prediction interval) could exceed these limits (Figures 2, 3 and 4). 

The simulation according to the “Generalizability Theory’ resulted in g-coefficients for the test in anteflexion of 0,99 (affected side), 0,96 (contralateral side), abduction 0,99 (affected side), 0,99 (contralateral side) and for the retest anteflexion: 0,99 (affected side) 0,98 (contralateral side) and abduction 0,99 (affected side), 0,99 (contralateral side) respectively.

**Figure 5:**
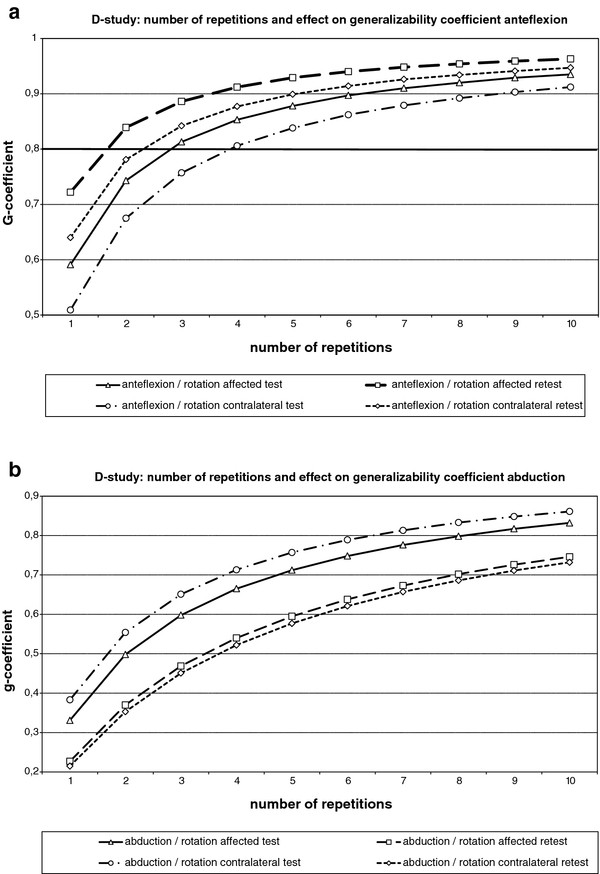
D study results with related rotations.

The performed D-study resulted in g-coefficients of 0,97 after 2 repetitions (anteflexion affected side), 0,91 after 2 repetitions (anteflexion contralateral side), 0,99 after 2 repetitions (abduction affected side), 0,97 after 2 repetitions (abduction contralateral side), 0,98 after 2 repetitions (anteflexion retest affected side), 0,96 after 2 repetitions (anteflexion retest contralateral side) and 0,98 after 2 times for both affected and contralateral side in the retest of abduction. (Figure 5a) Analysis of the combination of anteflexion and related rotations in a simulation of the generalizability theory resulted in less good reproducibility coefficients. In particular the reproducibility of abduction was largely influenced by the related rotations (Figure 5b).

## Discussion

The three-dimensional gyroscope under study showed good to excellent reproducibility for measuring anteflexion and abduction. An average of 2 repetitions was needed for a sound reproducibility of anteflexion and abduction measurement, In case the related rotations were imputed in the generalizability analysis, 2 up to 4 repetitions were needed for anteflexion movement, where 7–10 repetitions were needed for abduction. These differences might be explained by the fact that there is more variability in rotation during abduction compared to anteflexion. Mean differences for anteflexion and abduction between test and retest were −6 degrees (affected side), 9 degrees (contralateral side) and 15 degrees (affected side), 10 degrees (contralateral side) respectively. Bland Altman plots showed a level of agreement with a confidence interval for overall mean differences of −6 up to 15 degrees.

*Accordingly to El-Zayat et al*.*., the device related measurement error (based on 95 %-prediction intervals) could vary between −0.77 and 2.25 degrees depending on velocity of motion and the distance of the device to the centre of rotation. Because of the repeated measurements for each trial, we considered the possible effect of sensor error on the results as negligible*[14]*.*

A clinical anteflexion movement consists of a movement in the frontal plane with accompanying rotations of the humerus. The abduction movement consists of a movement in the sagital plane with accompanying movement of the humerus. With the acquired data we were able to calculate rotational movement during anteflexion and abduction. We therefore were able to assess the combined effect of movement in the frontal and sagital plane with accompanying rotations in a generalizability theory model.

In our study we focused on the reproducibility of testing. The results of testing were displayed in degrees; the validity of these results however needs further study.

During the study data had to be sent to Mc Roberts (Meanwhile the procedure has been improved and results can be processed in the outpatient clinic).

The reproducibility coefficients found in our study ranging from 0,96 to 0,99 are excellent compared to other measurement techniques, taking into account that the tests were performed in patients with different underlying shoulder pathology [3,6,8,12,13,19].

In this study we could prove that the tri-axial gyroscope is a reproducible instrument in the measurement of shoulder anteflexion and abduction in patients with different underlying pathologies.

## Conclusions

ROM is an essential measure in the diagnosis of shoulder impairments [1]. Several methods have been developed for the measurement of ROM [2,3]. These methods have poor reproducibility.[12,13]. The technique of a tri axial gyroscope could be a quick and simple method for the recording of three-dimensional shoulder movements. Our findings support the excellent reproducibility of a tri axial gyroscope for measurement of shoulder anteflexion and abduction.

In our study patient perceived pain showed to be of influence on the measurements carried out. The differences in VAS and DASH score might be explained by difference of underlying pathology, osteoarthritis and subacromial impingment, between groups. The differences in shoulder pathology however did not alter the reproducibility of testing.

Simulation according to the “Generalizabilty Theory” showed in a D-study that measurements only have to be repeated twice for reproducible results.

## Competing interests

The authors declare that they have no competing interests.

## Authors’ contributions

LIFP participated in the design of the study carried out the data collection and measurement, performed the statistical analysis and drafted the manuscript. NAG, RAdB and GHIMW participated in the design of the study and helped to draft the manuscript. All authors read and approved the final manuscript.

## Authors’ information

LIFP is currently positioned at the Sint Maartenskliniek, Nijmegen, The Netherlands, as an orthopedic surgeon specialized in upper extremity.

NAG is positioned at Delft University, Delft, The Netherlands as Program Director Health RAdB is positioned at the Department of Epidemiology of Maastricht University, Maastricht, The Netherlands as Professor of Physiotherapy.

GHIMW is Emeritus Professor in Orthopedic Surgery, Orthopaedic surgeon.

## Pre-publication history

The pre-publication history for this paper can be accessed here:

http://www.biomedcentral.com/1471-2474/13/135/prepub
